# New method for early evaluation of clitoris innervation using clitoro-perineal reflex after feminizing genitoplasty in early childhood: a pilot-study

**DOI:** 10.1038/s41598-021-86434-5

**Published:** 2021-03-29

**Authors:** Valeska Bidault, Nathalie Botto, Annabel Paye-Jaouen, Juliane Leger, Éliane Josset-Raffet, Laetitia Martinerie, Matthieu Peycelon, Alaa El-Ghoneimi

**Affiliations:** 1 Department of Pediatric Surgery and Urology, University Children Hospital Robert-Debré, APHP, University of Paris, 48, Bd Sérurier, 75935 Paris Cedex 19, France; 2 Centre de Référence Maladies Endocriniennes de La Croissance Et du Développement (CRMERC), Paris, France; 3 Department of Pediatric Endocrinology, University Children Hospital Robert-Debré, APHP, University of Paris, Paris, France

**Keywords:** Paediatric urology, Urogenital diseases

## Abstract

A major complication of feminizing genitoplasty in children is the loss of clitoral sensation with serious impact at adult life. We suggest a new method to evaluate the surgical results during childhood based on the bulbocavernosus or clitoro-perineal reflex (CPR). The afferent pathway of CPR implies the intact sensory receptors on the clitoral glans. Girls with congenital adrenal hyperplasia who were followed-up medically without surgery or who underwent feminizing genitoplasty with or without clitoroplasty were included (2002–2018). All clitoroplasties were standardized reduction clitoroplasty with preservation of neurovascular bundles associated with vaginoplasty and vestibuloplasty. Standardized examinations were prospectively performed including the CPR starting at one year postoperatively. The reflex was triggered by gentle touch of the glans by a cotton swab. Contraction of the perineal muscles was considered positive. Thirty-two children were operated at a median age of 8.6 months (5.8–12.1). Median follow-up (FU) was 3.9 years (1.3–6.4). Twenty-four patients had clitoroplasties: 17 were tested for CPR at one-year FU, and all had a positive test. Eight girls had genitoplasty without clitoral surgery, two of them were tested and were positive. Ten patients were managed without surgery, two of them were tested for the CPR and were positive. The reflex was always triggered easily and repeated at least twice during the FU. The clitoro-perineal reflex is a simple, non-invasive and reproducible test in early childhood and may serve as an early evaluation tool of clitoral innervation after feminizing genitoplasty. These results need to be confirmed at long term and completed at adult life.

## Introduction

Congenital adrenal hyperplasia (CAH) is the most frequent DSD (Differences of Sex Development) in the 46XX group. Surgical management usually includes feminizing genitoplasty (vaginoplasty and vestibuloplasty with or without clitoroplasty).

Clinical practice guidelines for CAH suggest that clitoral and perineal reconstruction for females with severe virilization of external genitalia could be considered in infancy and performed by an experienced surgeon^1^. Considering the multiple clinical presentations of DSD and the complex discussions these situations may sometimes raise among specialists, there is no consensus regarding the indications, the timing, the procedure, and the evaluation of outcomes of DSD surgery^2,3^. Given the lack of strong evidence for standardized surgical management in DSD situations, multidisciplinary expert teams should advocate for building collaborative and prospective surgical procedures with standardized consensual evaluation protocols^4,5^. Taking in consideration these concerns, the need for an evaluation tool adapted to young children becomes evident^6^.

However, there are debates regarding the optimal timing of surgery^7–9^. In the past 20 years, understanding of the anatomy and the neuroanatomy of the female genitalia has expanded greatly. Considerable advances in surgical techniques have emerged concomitantly, with the aim of improving the functional, cosmetic, and psychological results of this surgery. Long-term evolution of operated genitalia will occur at adult age more than 20 years after the surgical procedures^10^, and meanwhile methods may have changed several times during this period of time^11^.

Clitoroplasty may disrupt neurological pathways and compromise clitoral sensation, thus affecting sexual function. Very few studies have investigated the functional effects of feminizing genitoplasty. Most of them are based on a Sexual Function Questionnaire. Crouch et al. started to evaluate the effects of surgery on genital sensitivity by assessing sensitivity thresholds for the clitoris with a GSA (Genital Sensitivity Testing, GenitoSensory Analyzer)^12^. The GSA consists of two separate probes, including one for temperature measurements and one for vibratory testing, with a feedback patient response switch^13^.

But to date we do not know how to evaluate this function precociously after surgery in childhood.

Clitoral anatomy has been thoroughly investigated, in particular by Baskin et al.^14^. They have at first described clitoral innervations, showing that large bundles of nerves constitute a neuronal network along the corpus cavernosa with the highest density of fibers on the dorsal side of them. These bundles end up at the basis of the glans by perforating branches entering the glans to provide sensitivity until the tip of it.

**Figure 1:**
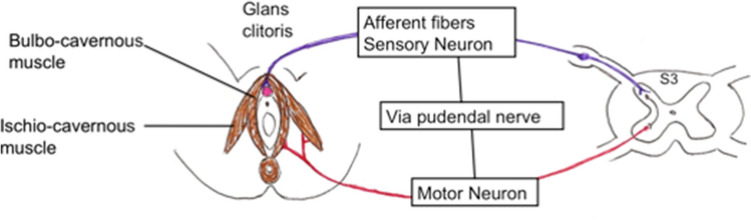
Schematic illustration of the clitoro-perineal Reflex.

We can assess clitoral sensitive innervation using a system of monosynaptic reflex. The bulbocavernosus reflex is considered one of the sacral neurophysiological tests of the greatest clinical utility. It is a monosynaptic sensory-motor reflex mediated through the pudendal nerve (Fig. 1). The afferent somatic sensory pathway starts in the clitoris with the dorsal clitoral nerve, and then the neurological influx progresses along the pudendal nerve to the sacral plexus and finally reaches the sacral roots S2, S3 and S4. Along the efferent somatic motor pathway information is sent through Onuf’s nucleus neurons, sacral roots S2, S3 and S4, the sacral plexus, the pudendal nerve (deep motor branch) and the bulbo-cavernous muscle^15^. Bors and Blinn first used this reflex for the examination of the neurogenic bladder^16^. It was then studied on an electrophysiological basis in 11 male volunteers^17^. The bulbocavernosus reflex was even studied specifically in young boys^18^.

With full background knowledge, reduction of the clitoris in feminizing surgery should not violate the extensive innervation that predominates on the dorsal aspect of the glans.

We suggest a new method to evaluate the surgical results of clitoroplasty in childhood, based on the bulbocavernosus reflex. We propose the term of Clitoro-Perineal Reflex (CPR) here, as our study is focused on the clitoral innervaton.

## Materials and methods

Children who were diagnosed and followed-up in our institution for CAH during the period between 2002 and 2018 were included. The study was performed after approval by the local Ethics Committee of the University Hospital Robert-Debré and declared at the National Committee of Informatics and Freedom (authorization #1743855v0). Written informed consent was obtained from the parents. Written informed consent for photography use for research was also obtained for each photography shown in the manuscript.

All methods were carried out in accordance with relevant guidelines and regulations.

**Figure 2:**
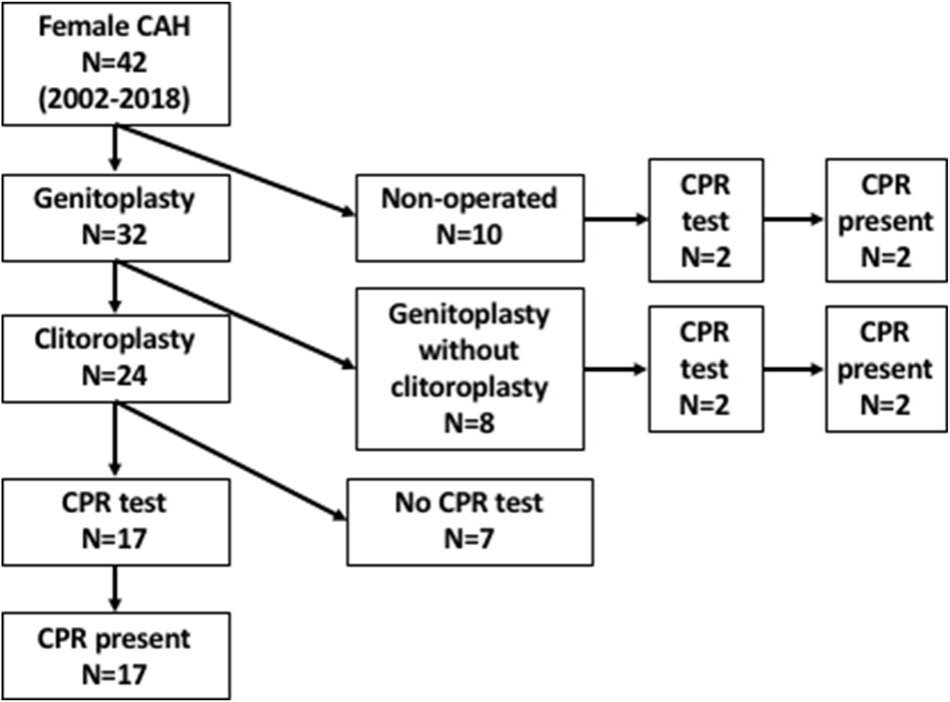
Flow chart of the study population. *CAH* congenital adrenal hyperplasia, *CPR* Clitoro-Perineal reflex, *N* number.

Three groups of patients were identified for the aim of this study (Fig. 2): Group 1, children who underwent feminizing genitoplasty including reduction clitoroplasty during the first two years of life; Group 2, children who underwent feminizing genitoplasty (vestibuloplasty and vaginoplasty) without reduction clitoroplasty, and; Group 3, children managed medically without any surgery.

The decision to perform or not a clitoroplasty (in Groups 2 and 3) was taken according to the degree of virilization of the external genitalia, and the surgeon’s and parents’ common agreement and preferences. Two children from each group without clitoral surgery were included in the study to analyze the reflex in non-operated “control” children with CAH.

All patients who had a total feminizing genitoplasty (Group 1) had standardized reduction clitoroplasty with preservation of the neurovascular bundles associated with vaginoplasty and vestibuloplasty. The procedure was conducted under magnifying loops, without tourniquet. The shaft of the clitoris was incised at the ventral surface, then the dissection started beneath the Buck’s fascia. The neurovascular bundles were kept intact to the deep surface of the fascia without individual dissection. After a full-length separation of the Buck’s fascia from the dorsal and lateral surfaces of the corpus cavernosa, the corpora were ligated at their base by transfixing resorbable sutures. The corpus cavernosa bodies were resected from the base to the glans. If the volume of the glans was significant, the corpora were removed totally, otherwise a cushion of corpora was left intact inside the glans. The glans was re-inserted directly to the tunica albuginea of the stump by four quadrants resorbable sutures. None of children had glans reduction surgery.

**Figure 3:**
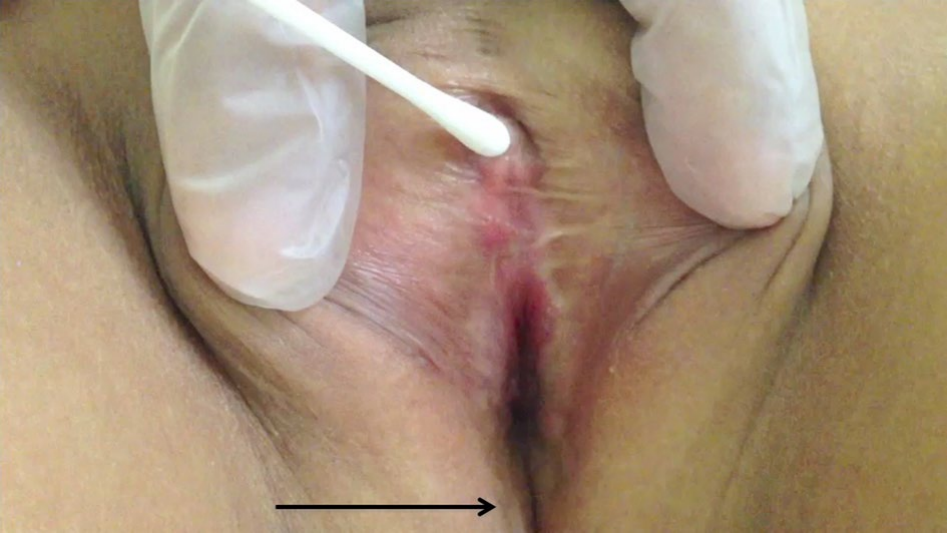
Description of the clitoro-perineal reflex. The reflex was triggered by gentle touch of the glans by a cotton swab. Contraction of the perineal muscles was observed (Arrow). The Video shows how the reflex is triggered with clear observation of perineal muscles contractions, one year after feminizing genitoplasty including clitoral reduction (Video clip).

Standardized examinations were prospectively performed by a senior pediatric urologist. Patients who were anxious by perineal examination were proposed nitrous oxide sedation at the outpatient clinic. The morphological aspects of genitalia were described in detail. The external masculinization score (EMS) was used^19^. The CPR was triggered by gentle touch of the clitoral glans with a cotton swab at the outpatient clinic and was tested after one year of surgery and during the regular follow-up (FU). The cotton swab is kept at the room temperature. The result was considered positive if there were contractions of the perineal muscles (bulbocavernosus and ischio-cavernosus muscles) (Fig. 3).

Descriptive statistics were performed using Fisher’s exact test for categorical variables, and the Mann–Whitney test for non-parametric continuous data (median and IQR are presented). Variables were analyzed on univariate analysis with a *p* value of 0.05 considered statistically significant (Stata 12.1; StataCorp, College Station, TX, USA).

### Ethics approval

The study was performed after approval by the local Ethics Committee of the University Hospital Robert-Debré and declared at the National Committee of Informatics and Freedom (authorization #1743855v0). Written consent for photography use for research was obtained for each photography shown in the manuscript and signed by the parents.

## Results

A total of 42 patients with CAH was identified and followed-up during the study period. Descriptive characteristics of the cohort is summarized in Table 1. Thirty-two children were operated during their first 2 years of life at a median (IQR) age of 8.6 months (5.8–12.1). Median FU was 3.9 years (1.3–6.4). Among the 24 patients who had clitoroplasty (Group 1), 17 were tested for CPR at one-year FU. All patients (100%) had a positive test. The CPR was triggered easily and repeated at least twice during the FU. All had a pinkish and well-vascularized glans, and well perceived perineal contractions. Seven patients were overseas patients and were not tested for CPR as the FU was not done on site.

**Table 1:** Descriptive characteristics of the cohort.

	Genitoplasty with clitoroplasty (Group 1) N = 24	Genitoplasty without clitoroplasty (Group 2) N = 8	*p* value
Median (IQR) age at surgery (mos.)	8.4 (5.5–12.1)	8.6 (6.9–12.8)	0.601
Median (IQR) preoperative clitoris length (mm)	25.0 (22.8–30.0)	17.5 (13.8–20.5)	0.0005
Median preoperative EMS	6.0 (3.0–6.0)	3.0 (3.0–3.0)	0.003
Median (IQR) follow-up (yrs.)	4.0 (0.7–9.7)	3.4 (2.7–4.7)	0.878

*EMS* external masculinization score, *IQR* interquartile, *mm* millimeter, *mos.* Months, *N* number, *yrs.* years.

Group 2 included eight girls who had genitoplasty without clitoral surgery. All of them were managed after 2012. The CPR was tested in two and both had a positive test.

During the same period, ten girls with CAH were managed without any surgery (Group 3) with a median FU of 7.8 years (5.7–12.5). Two of them were tested for CPR at clinics and were positive.

The global results of the cohort are summarized in Fig. 2.

## Discussion

Feminizing genitoplasty in children is one of these surgeries that demonstrate the complexity of management and outcome evaluation in a rare congenital anomaly. Many articles recommend achieving standardization in surgical practice. Management of these complex cases should be performed by experienced professionals in multidisciplinary settings^7^.

The optimal evaluation is certainly at long-term when the patient starts an active sexual life. Meanwhile, this fact reduces the benefits of these long-term studies, as the time laps between the surgery and the evaluation is too long. Most often the surgical techniques and concepts quickly change over time, underlying the expected answers from surgeons that the study was done using old methods and cannot be used to criticize the current modern method. This endless discussion may lead to the loss of benefits that the patients may have had if an early evaluation was feasible even if it was still suboptimal.

Clitoral surgery has evolved from clitoral amputation to more conservative procedures. Clitoral reduction surgery with preservation of the neurovascular bundles is now the standard procedure for feminizing genitoplasty^20^. All described modern techniques published in the last 20 years are based on a common principle to preserve the neurovascular bundles to keep a well-vascularized and sensitive glans. The ideal aim is to preserve the future sexual function of the operated children. Variation of the procedures is from the original description by Goldwyn^21^ to the removal of the spongy erectile tissue within the corpora^22^ or more corporeal sparing dismembered clitoroplasty recently described by Pippi Salle et al.^20,23^.

Long-term results on clitoral sensitivity and sexual functions are controversial and frequently biased by the large rate of loss to FU from the original cohort of patients.

In the survey of Yankovic et al.^7^, outcomes from clitoroplasty were reported as very good or satisfactory by 84% of the delegates. Most of the time, however, this evaluation is primarily cosmetic and does not consider clitoral innervation.

Crouch et al. have reported in 2004 a long-term FU of genital sensation and sexual function affected after clitoral surgery^12,24^. In the significant publication by Crouch et al. they compared the genital sensation in women with CAH (with and without surgery) and normal controls. The GSA (Genitosensory Analyzer) was used to assess sensitivity thresholds. To assess sexual function, a questionnaire incorporating GRISS (Golombok Rust Inventory of Sexual Satisfaction) was used. Results showed decreased sensitivity to warmth, cold and vibration in women with CAH who had surgery. This study demonstrated that surgery directly impacted clitoral sensitivity, but it was carried out on adult women operated in the early eighties and this series included many of patients (6/15) with clitoral amputation. Therefore, the conclusion supported by this article cannot be generalized to apply to more recent surgical techniques. Moreover, the study was carried out on only 15 adults among a cohort of 52 adults operated in childhood. Recently, an equivalent study was done by Lesma et al.^25^, and showed that although clitoral sensitivity in sexually active patients with CAH treated with Passerini-Glazel feminizing genitoplasty was significantly reduced compared to controls, sexual function in those patients was not statistically or clinically different from their healthy counterparts. In this recent study, only 10% of the operated patients could be evaluated at long term. These controversial results encouraged us to find a method to evaluate, in early childhood, the impact of the surgery on clitoral innervation.

Sensory evaluation tests have been completed and described with pudendal evoked potentials during feminizing genitoplasty^26^. This study showed that modern procedures of genital reconstruction allow the preservation of nerve conduction in the neurovascular bundles.

It is certain that clitoral sensitivity and its sexual function cannot be reduced to a simple sensory-motor reflex test. The neuroanatomic study of female perinea is indeed very complex. There are numerous branches, with intervention of the autonomic nerves system^27^.

The literature is already rich of publications on the female genital innervation with somatosensory evoked potentials (SEP). The best evaluation would be to perform pudendal SEP in every young girls operated with clitoroplasty, with electrodes placed on the clitoris for stimulation, and electrodes placed on the scalp for recording neuronal action within the brain^28,29^ However, this evaluation requires a specialized technical platform with dedicated technicians, and may be time-consuming.

Numerous tests are listed to evaluate tactile and erogenous sensitivity in adults, in particular after sex reassignment surgery, with the measurement of pressure and vibration, in g/mm and microm respectively^30^. Again, these tests are only feasible in adolescents and young adults, but not during childhood, even if most of expert teams in pediatric urology perform surgery in early childhood.

With this simple testing of the clitoro-perineal reflex, we get an idea of the clitoral sensitive innervation, even if a normal sensory track alone is not predictive of normal sexual function in the future.

CPR is simple to assay during physical examination, even in young girls, providing that the surgeon can gain confidence of the patient for the perineal testing. It can be therefore repeated easily by the surgeon during the post-operative FU until the patients reach sexual maturity and functional evaluation of the clitoris only then could be done.

We acknowledge that there are some limits in our study. The CPR investigates only the simple reflex effects without any higher perception of sensitivity. We need also to continue to follow-up these patients until adulthood. A standard test should be performed and compared to the reflex results in childhood. Moreover, it is not a quantitative test and we could not establish an objective way to quantify visually the contraction of the muscles. In our current study, we did not define a threshold to activate the CPR neither the effects of external factors as temperature on the CPR activation. In fact, our method to trigger the reflex was chosen as the least invasive to the child to be able to use it during the postoperative clinical examination. It is important to follow a standard method as described and shown in Fig. 3 and Video supplement: the CPR is triggered by gentle touch of the clitoral glans with a cotton swab (kept at the room temperature).

Our simple message is that by this non-invasive method feasible in young children we may know that the current modern technique of feminizing genitoplasty does not disrupt the afferent nervous pathway of clitoral innervation. Our perspectives are to reevaluate these girls after puberty and sexual life onset, to compare our results on pre-pubertal CPR to the final clitoral sensation and function.

## Conclusion

The clitoro-perineal reflex is a simple, non-invasive, and reproducible test. It is feasible in early childhood and may serve as an early evaluation tool of clitoral sensitive innervation after feminizing genitoplasty. Certainly, these results need to be confirmed at a long-term follow-up and completed at adult life by a complete evaluation of the clitoral sensory thresholds.

## Supplementary Information

Supplementary Video 1.

## Abbreviations

CPR: Clitoro-perineal reflex
FU: Follow-up
CAH: Congenital adrenal hyperplasia
DSD: Differences of sex development
EMS: External masculinization score
IQR: Interquartile
mm: Millimeter
mos.: Months
N: Number
yrs.: Years
